# Differences in school environment, school policy and actions regarding overweight prevention between Dutch schools. A nationwide survey

**DOI:** 10.1186/1471-2458-10-42

**Published:** 2010-01-28

**Authors:** Salome Scholtens, Lideke Middelbeek, Suzanne I Rutz, Goof Buijs, Wanda JE Bemelmans

**Affiliations:** 1 Centre for Prevention and Health Services Research, National Institute for Public Health and the Environment (RIVM), P.O. Box 1, 3720 BA, Bilthoven, The Netherlands; 2 The Netherlands Health Care Inspectorate, P.O. Box 20584, 1001 NN, Amsterdam, The Netherlands; 3 The Netherlands Institute for Health Promotion and Disease Prevention (NIGZ), P.O. Box 500, 3440 AM, Woerden, The Netherlands

## Abstract

**Background:**

Schools are regarded as an important setting for the prevention of overweight. This study presents a nationally representative picture of the obesogenity of the school environment, the awareness of schools regarding overweight, and actions taken by the schools aiming at overweight prevention. In addition, differences between school levels were studied.

**Methods:**

In 2006-2007, questionnaires were sent to all Dutch secondary schools (age group 12-18 years). Prevalences of the outcome variables were calculated for the schools in total and by school level. The association between school level and outcome variables were analysed by a log linear regression.

**Results:**

Unhealthy foods and drinks are widely available at secondary schools. One third of the schools indicated that overweight has increased among students and half of the schools agreed that schools were (co)responsible for the prevention of overweight. Only 3% of the schools have a policy on overweight prevention. Small differences were observed between vocational education schools and higher education schools. The presence of vending machines did not differ by school level, but at vocational education schools, the content of the vending machines was less healthy.

**Conclusion:**

This study describes the current situation at schools which is essential for the development and evaluation of future overweight prevention policies and interventions. In general, secondary schools are not actively involved in overweight prevention and the nutritional environment at most schools could be improved. The small differences between school levels do not give reason for a differential approach for a certain school level for overweight prevention.

## Background

The prevalence of overweight and obesity among children is increasing throughout the world[1]. In the Netherlands, 14.5% of the boys and 17.5% of the girls aged 4-15 years were overweight or obese in 2003, compared to 3.9% and 6.9% in 1980 respectively[2]. An 'obesogenic' environment is described as a crucial factor in the development of overweight among children and therefore provides opportunities for overweight prevention[3,4]. Since children spend a significant part of their time at school, the school is regarded as an important setting that could influence the development of overweight[5,6]./

Overweight is inversely related to socio-economic status (SES) and the difference in overweight prevalence between different SES groups already starts to develop during school age[7]. Children of parents with a low SES are more at risk to become overweight compared to children with parents with a higher SES[8,9]. In general, the number of years of education or the highest level of education completed is often used as a proxy for SES.

The Dutch secondary educational system (for children from the age of 12 years until 18 years) consists of three different types of educational levels, namely: Preparatory vocational education (4 years); Senior general education (5 years); University preparatory education (6 years). Some secondary schools offer only one of the three levels, while others offer all three levels (mixed schools). Children who follow the 'preparatory vocational education', in general, reach a lower educational level and engage practical, lower paid jobs later in life than students who graduate from the other two levels. Attending a certain type of school during teenage years can therefore be seen as an early indicator for the probable SES later in life.

Studies in the Netherlands showed that students engaged in preparatory vocational education were more likely to be overweight than students in higher educational levels[10,11]. Besides differences in the home environment and in individual factors, differences in the 'obesogenity' of the school environment may contribute to differences in overweight prevalence among students of vocational and higher education schools.

For the design and implementation of prevention strategies it is important to gain insight in the current situation regarding the environment at secondary schools. Therefore, a national survey was conducted in 2007 in The Netherlands on several issues related to the prevention of overweight at schools.

The first aim of this study is to present a nationally representative picture of the obesogenity of the school environment, the awareness of schools regarding overweight, and school health policy and specific actions regarding overweight that have been undertaken by the schools. A secondary aim is to investigate differences between the different school levels. This latter information is important in the context of counteracting socioeconomic differences in health and adds insight in explanatory mechanisms for the positive association between SES, which is often defined by educational level, and overweight.

## Methods

### Study design

This study was performed within the scope of a national survey on the current nutritional and physical environment at Dutch secondary schools. The survey was conducted through a postal questionnaire by the National Institute for Public Health and the Environment together with the Netherlands Institute for Health Promotion and Disease Prevention, financed by The Netherlands Health Care Inspectorate and co-financed by the Dutch Ministry of Health.

The first mailing was sent to all secondary schools of the Netherlands in November 2006 and a second mailing was performed in January 2007 to the schools that had not responded so far to increase the total response. Together with this second mailing a non-responders card was sent to the schools in which the reason for non-response was queried.

In the Netherlands, the majority of the secondary schools consist of different sites. At the time of the survey there were 577 secondary schools consisting of 1250 school sites. To increase the readability of this paper from now on 'school sites' will be referred to as 'schools'. The educational level of the schools was not known on beforehand, but was questioned in the questionnaire.

Of the 1250 approached schools, 555 (44%) completed the questionnaire, from which 359 (66%) responded on the first mailing and 196 (34%) on the second. The non-response card was returned by 148 (12%) schools. Thirty-three schools indicated in the questionnaire that they offered only individual education or special education. These schools were excluded from the analysis, because the educational system of these schools differs from the regular secondary schools. For another seven schools the school level was missing, leaving 515 schools available for analysis.

For this study, no ethical approval was necessary according to the Dutch Central Committee on Research involving Human Subjects http://www.ccmo.nl because the questionnaires were not directed at children, no direct health related questions had to be answered and no medical investigation were included.

### Questionnaire

The questionnaire consisted of 80 questions divided in six parts (Table 1). The questionnaire started with questions on general characteristics of the schools. The second part consisted of questions on the school environment, for instance the presence and content of vending machines and the possibilities for the students to be physically active during lunch breaks. The third part consisted of questions on health education, the fourth on school projects on overweight prevention and the fifth on the school's health policy. The questionnaire concluded with questions regarding who completed the questionnaire and whether the questions were clear and easy to answer.

**Table 1:** Content of the questionnaire

Part	Topic	Sub topics	Number of questions	Example questions
1	General characteristics		5	- What is the total number of students at your school? - Which educational levels are offered at your school?
2	The school environment	- Inside the school building - The canteen - At and around the school property	28	- Are there soft drink vending machines present at school? - How would you describe the proportion of high caloric and low caloric drinks in the soft drink vending machine?
3	Health education*	- Biology - Physical activity education - Health and hygiene classes	15	- How many hours of physical activity education do the children receive per week?
4	Participation in projects		12	- Did your school participate in one of the following national projects in the last two years?
5	School policy	- General health policy - School policy regarding: Nutrition Sports/physical activity Overweight	17	- Does your school have a general health policy? - Which topics receive special attention within the general health policy?
6	Closing questions		3	- What is the professional function of the person who completed the questionnaire?

*These questions with regard to the curriculum were not analysed in this study, because within a school level the curriculum is fairly similar between schools. All students in the Netherlands have to pass the same final exam for graduation, depending on the school level.

For this study, the questions relevant for the study question were selected from the questionnaire. In some cases, questions were combined to one outcome variable. In total, 45 outcome variables were defined describing the school environment, awareness and responsibility of the schools towards the overweight problem, and the school policy and actions taken by the school to prevent overweight.

### Data Analysis

The school level was categorized in three categories: 1) vocational education schools, i.e. schools only offering 'preparatory vocational education'; 2) mixed schools, i.e. schools offering 'preparatory vocational education' and 'senior general education' and/or 'university preparatory education'; 3) higher education schools, i.e. a combination of schools offering 'senior general education' and/or 'university preparatory education'.

The Netherlands can be divided into 33 municipal health services regions. The municipal health services are responsible for implementation of the preventive health care in the region. Schools in different regions could therefore differ from each other with regard to the school health policy or participation in projects on overweight prevention due to differences between municipal health services in overweight prevention policy and the presences of projects. Prior to the analyses on the school characteristics, a multi level analysis was conducted to see whether the schools within the 33 municipal health services regions differed from each other. We used the NLMIXED procedure in SAS.

Prevalences of the outcome variables were calculated for the schools in total and by school level. The association between the school level and the outcome variables were analysed by a log linear regression, because the probability of most outcome variables was relatively high. Vocational education schools and mixed schools were compared with the higher education schools. All associations were adjusted for school size. In addition, the interaction between school level and school size was tested. The school size was defined as the total number of children attending the school and divided in less than 500 students, 500 to 1000 students and over 1000 students. Data analysis was conducted using SAS software version 9.1 (SAS Institute, Inc., Cary, NC, USA). P-values below 0.05 were considered to be statistically significant.

## Results

### Study characteristics and reasons for non-response

The main reason the schools indicated in the non-response card for not participating in the study (n = 148) was that they could not participate in every study conducted in secondary schools (72%). One third indicated that they did not have time to complete the questionnaire.

The prevalence of the presence of a soft drink vending machine or a vending machine containing sweets and candy bars at school did not differ between the schools that completed the questionnaire and schools that only returned the non-response card. In both groups, 88% of the schools reported that there was a soft drink vending machine present at school and about 76% of the schools reported that there was a vending machine containing sweets and candy bars. Of the schools that completed the questionnaire 14% had a policy on overweight prevention compared to 31% of the schools that only complete the non-response card.

Forty-two percent of the schools (n = 216) were vocational education schools, 45% (n = 232) were mixed schools and 13% (n = 67) were higher education schools. The vocational education schools were in general smaller (mean = 604; sd= 501) than the mixed schools (mean = 1006; sd= 660) and the higher education schools (mean = 1101; sd= 620). The median school size was 700 students. 180 schools had less than 500 students, 165 schools had between 500 and 1000 students and 170 schools had over 1000 students.

Of the schools that participated in the study, 95% (n = 472) indicated that the questions in the questionnaire were clear and easy to answer. The percentage of missing data on individual questions was small. Most questionnaires were completed by more than one person (55%, n = 286). Seventy-one percent of the questionnaires (n = 358) was (co-) completed by the principal or the (assistant) manager of the school, 49% (n = 247) was (co-) completed by a teacher in biology, in physical activity, or in health and hygiene, and 24% (n = 123) was (co-) completed by an employee of the canteen at school.

### School environment

At the majority of the schools a soft drink vending machine (91%, n = 446) and/or a vending machine containing sweets and candy bars (81%, n = 413) is present (Table 2). At 78% (n = 393) of the schools there is a supermarket, gas station or a fast food restaurant in the neighbourhood (within 1 km of the school). At 68% (n = 345) of the schools there are facilities at or around the school property where the students can be physically active, for example a soccer field or a basketball field.

**Table 2:** The school environment

	Total	School level				
		**Vocational education schools**	**Mixed schools**	**Higher education schools**	**Vocational education schools versus higher education schools**^#^	**Mixed schools versus higher education schools**^#^
	**n = 515**	**n = 216**	**n = 232**	**n = 67**		
	
	**% (n)**	**% (n)**	**% (n)**	**% (n)**	**OR (95%CI)**	**OR (95%CI)**

Soft drink vending machine present at school	91.4 (466)	90.2 (194)	91.3 (209)	95.5 (63)	1.0 (0.9;1.1)	1.0 (0.9;1.0)

Percentage of soft drink vending machines present at school that contain light soft drinks	79.8 (372)	75.4 (147)	82.2 (171)	85.7 (54)	0.9 (0.8;1.0)	1.0 (0.9;1.1)

Soft drink vending machines contain more unhealthy drinks than healthy drinks	57.9 (268)	61.9 (120)	58.5 (121)	43.6 (27)	1.4 (1.0;1.9)	1.4 (1.0;1.9)*

Vending machine present at school that contains sweets/candy bars	80.7 (413)	75.6 (161)	84.9 (197)	82.1 (55)	1.1 (0.9;1.2)	1.1 (1.0;1.2)

Sweets/candy bars vending machines contain more unhealthy than healthy foods	63.7 (260)	70.6 (113)	64.4 (125)	40.7 (22)	1.7 (1.2;2.4)*	1.6 (1.1;2.3)*

There is a supermarket, gas station, or fast food restaurant in the neighbourhood of the school	78.3 (393)	76.7 (161)	79.7 (181)	78.5 (51)	1.1 (0.9;1.2)	1.1 (0.9;1.2)

The students are allowed to leave the school property during school hours	57.4 (295)	42.6 (92)	65.8 (152)	76.1 (51)	0.6 (0.5;0.8)*	0.9 (0.8;1.0)

There are facilities at and around the school property where the students can be physically active	68.1 (345)	63.1 (135)	70.3 (161)	76.6 (49)	0.8 (0.7;1.0)	0.9 (0.8;1.1)

^# ^Associations are adjusted for school size

* p < 0.05

The vocational education schools did not differ from the higher education schools with regard to the presence of vending machines and a canteen, but the vending machines and the canteen contained a less favourable selection of foods and drinks. The vocational education schools indicated more often that the vending machines and the canteen contained more unhealthy foods and drinks than healthy foods and drinks. Vocational education schools had fewer facilities at and around the school property to be physical than the higher education schools. Most associations were attenuated after adjustment for school size. For example, the association between school level and content of the soft drink vending machines was OR = 1.42, 95%CI: 1.05-1.93 in the crude analysis and OR = 1.35, 95%CI: 0.99-1.85 in the adjusted analysis.

### Awareness and responsibility towards the overweight problem

One third of the schools (n = 168) agreed that the prevalence of overweight has increased among students at their school (Table 3), but 78% (n = 392) indicated that overweight was not more prevalent among the students of their school than among the 12 to 18 year old children in the general population. According to the schools, the parents of the students and the students, students themselves were principally responsible for the development of overweight among students. Less than half of the schools indicated that the schools were (co-) responsible for the development of overweight among their students.

**Table 3:** Awareness and responsibility of the schools towards the overweight problem

	Total	School level				
		**Vocational education** **schools**	**Mixed schools**	**Higher education schools**	**Vocational education schools versus higher education schools^#^**	**Mixed schools versus higher education schools^#^**
	
	**n = 515**	**n = 216**	**n = 232**	**n = 67**		

	**% (n)**	**% (n)**	**% (n)**	**% (n)**	**OR (95%CI)**	**OR (95%CI)**

The prevalence of overweight has increased among the students	33.3 (168)	38.9 (82)	30.0 (68)	27.3 (18)	1.4 (0.9;2.2)	1.1 (0.7;1.7)

Overweight is less prevalent among the students at school than in the general population	78.1 (392)	69.7 (145)	82.8 (188)	88.1 (59)	0.8 (0.7;0.9)*	0.9 (0.8;1.0)

Schools are responsible for the prevention of overweight among students	45.8 (233)	47.9 (102)	43.2 (99)	47.8 (32)	1.0 (0.7;1.4)	0.9 (0.7;1.2)

Parents are responsible for the prevention of overweight among their children	98.4 (501)	97.7 (208)	99.1 (227)	98.5 (66)	1.0 (0.9;1.1)	1.0 (0.9;1.1)

Students themselves are responsible for the prevention of overweight	84.5 (430)	80.3 (171)	87.3 (200)	88.1 (59)	0.9 (0.8;1.0)	1.0 (0.9;1.1)

The government is responsible for the prevention of overweight among students	22.2 (113)	24.4 (52)	21.8 (50)	16.4 (11)	1.6 (0.9;2.9)	1.4 (0.8;2.6)

^# ^Associations are adjusted for school size

* p < 0.05

The schools reported whether they thought that the students at their school were more often or less often overweight than the 12 to 18 year old children in the general population. Compared to vocational education schools higher education schools indicated more often that the students at their schools were less often overweight than the 12 to 18 year old children in the general population. No differences were observed between the school levels regarding the opinion on the responsibility for the development of overweight among students. Adjustment for school size did change the crude associations slightly, but did not affect our conclusions.

### School health policy and actions taken to prevent overweight

Only a small proportion of the schools reported to have a general health policy (16%, n = 81), a policy on healthy nutrition (15%, n = 75), or a policy on overweight prevention (3%, n = 16) (data not shown). 65% (n = 52) of the schools with a general health policy indicated that nutrition, physical activity or overweight prevention had a priority within the general health policy. No differences between school levels were observed with regard to a general health policy, a policy on healthy nutrition, or a policy on overweight prevention.

The schools indicated more often that they had actions taken to stimulate healthy eating behaviour (66%), to discourage unhealthy eating behaviour (85%), or to stimulate physical activity (74%) than actions specifically aiming at the prevention of overweight (24%) (Table 4). The actions that were most often mentioned were: it is forbidden to sell certain unhealthy foods in the canteen at school, for instance deep-fried foods (36%, n = 180); the content of the vending machines has been changed so that the vending machines now also contain healthy products (41%, n = 206); the school organises often after-school activities where the students can be physically active (60%, n = 303). In general, vocational education schools and mixed school did not differ from higher education schools with regard to whether or not the school had taken actions. On the level of individual actions, not many differences were observed between school levels. Vocational educational schools were significantly less likely to have forbidden the sale of certain unhealthy foods in the canteen than higher education schools. Also, vocational educational schools indicated less often that they organised often after-school activities where the students could be physically active. Vocational education schools had more often guidelines in place to identify and to help students with overweight.

**Table 4:** Actions taken to prevent overweight at school

	Total	School level				
		**Vocational education schools**	**Mixed schools**	**Higher education schools**	**Vocational education schools versus higher education schools^#^**	**Mixed schools versus higher education schools^#^**
	
	**n = 515**	**n = 216**	**n = 232**	**n = 67**		

	**% (n)**	**% (n)**	**% (n)**	**% (n)**	**OR (95%CI)**	**OR (95%CI)**

Actions taken to stimulate healthy eating behaviour^§^	66.4 (330)	62.0 (129)	67.7 (151)	75.8 (50)	0.9 (0.7;1.1)	0.9 (0.8;1.1)

Healthy products are made less expensive than unhealthy products	28.6 (142)	23.6 (49)	31.4 (70)	34.9 (23)	0.8 (0.5;1.2)	0.9 (0.6;1.4)

The canteen offers a wide variety of healthy foods	27.4 (136)	23.1 (48)	28.3 (63)	37.9 (25)	0.8 (0.6;1.3)	0.8 (0.6;1.2)

Actions taken to discourage unhealthy eating behaviour^§^	84.9 (428)	83.5 (177)	87.2 (197)	81.8 (54)	1.0 (0.9;1.2)	1.1 (0.9;1.2)

It is forbidden to sell certain unhealthy foods	35.7 (180)	29.7 (63)	38.1 (86)	47.0 (31)	0.7 (0.5;0.9)*	0.9 (0.6;1.2)

It is forbidden to consume certain unhealthy foods	4.0 (20)	3.3 (7)	5.3 (12)	1.5 (1)	2.0 (0.2;16.8)	3.3 (0.4;25.4)

Actions taken to stimulate physical activity^§^	73.9 (374)	68.4 (145)	79.0 (181)	73.9 (48)	0.9 (0.8;1.1)	1.1 (0.9;1.3)

The school stimulates the students to be physically active during breaks	19.8 (100)	17.5 (37)	21.8 (50)	20.0 (13)	0.7 (0.4;1.3)	1.1 (0.6;1.8)

The school often organises activities for the students to be physical activity after school hours	59.9 (303)	50.9 (108)	66.4 (152)	66.2 (43)	0.9 (0.7;1.1)	1.1 (0.9;1.3)

Actions taken to prevent overweight^§^	24.0 (108)	26.3 (50)	24.0 (49)	15.8 (9)	1.7 (0.8;3.2)	1.5 (0.8;2.9)

There are guidelines to identify and to help students with overweight	12.9 (58)	17.9 (34)	10.3 (21)	5.3 (3)	3.1 (1.0;10.2)	1.9 (0.6;6.3)

Students who are overweight get more attention during physical activity classes	13.3 (60)	12.1 (23)	14.7 (30)	12.3 (7)	1.0 (0.4;2.3)	1.1 (0.5;2.5)

The school participated in a national or regional project for overweight prevention	28.4 (145)	29.3 (63)	26.8 (61)	31.3 (21)	0.9 (0.6;1.5)	0.8 (0.5;1.3)

The school expects to pay more attention to overweight prevention in the future	59.4 (299)	64.6 (137)	57.8 (130)	48.5 (32)	1.3 (1.0;1.7)	1.2 (0.9;1.6)

^# ^Associations are adjusted for school size

^§ ^The school has taken at least one action

* p < 0.05

More than half of the schools expected to pay more attention to overweight prevention in the future. In particular the vocational education schools expected that the attention paid to overweight prevention would increase (64%, n = 137) compared to higher education schools (49%, n = 32).

In general, adjustment for school size did change the crude associations slightly, but did not affect our conclusions. However, the association between the individual actions to stimulate healthy eating behaviour and the school level attenuated considerably after adjustment for school size. For example, the association between school level and the action 'the canteen offers a wide variety of healthy foods' was OR = 0.61, 95%CI: 0.41-0.91 in the crude analysis and OR = 0.84, 95%CI: 0.56-1.27 in the adjusted analysis.

### School size and municipal health services region

The multi level analysis showed that there was no clustering by municipal health services region. Therefore, all schools were analysed together.

At small schools (<500 students, n = 180) a soft drink vending machine, a vending machine containing sweets and candy bars and/or a canteen was present less often compared to large schools (>1000 students, n = 170) and the vending machines contained a less favourable food and drinks selection. Also, small schools had fewer facilities at and around the school property where the students could be physically active. Whether or not the school had a general health policy, a policy on healthy nutrition or a policy on overweight prevention did not depend on the school size. In general, small schools had less often taken actions to stimulate healthy eating behaviour, to discourage unhealthy eating behaviour, or to stimulate physical activity than large schools. Small school indicated more often than large schools that parents were informed about the unhealthy eating behaviour of their children.

Because school size was associated with school level and with a number of outcome variables, all associations between school level and the outcome variables were adjusted for school size. The analyses were not stratified by school size, as the number of observations was too small to analyse the data for the different school sizes separately and the tests for interaction showed that the majority of the interactions were not statistically significant.

## Discussion

The prime contribution of this extensive monitor is that it shows the current situation at Dutch secondary schools, with regard to the obesogenity of the school environment, the awareness of the schools about the overweight problem and the readiness for action. This baseline measurement is essential for the development and the evaluation of future overweight prevention policy and interventions.

At this moment, unhealthy foods and drinks are widely available at the majority of the secondary schools or in the neighbourhood of the school. Schools seem to become aware of the overweight problem and their role in the prevention of overweight. However, the number of schools that have a policy on healthy nutrition or on overweight prevention was low. The majority of the schools have taken some actions to stimulate healthy eating behaviour and to increase levels of physical activity, but only a couple of schools indicated that they had taken actions specifically aiming at the prevention of overweight among students.

Small differences were observed between vocational education schools and higher education schools. The school environment of vocational education schools looked more obesogenic than higher education schools, because the content of the vending machines was less favourable at vocational education schools and there were fewer facilities for the students to be physically active. However, vocational education schools seemed to be more aware of the overweight problem than higher educational schools.

### Strength and limitations

Strength of the study is that the study shows a complete picture of the current situation at Dutch secondary schools. The response on the questionnaire was good and schools of different school sizes and school levels in all regions of the Netherlands were represented.

In the Netherlands, schools are often invited to participate in studies or to complete questionnaires. Therefore it is difficult to motivate schools to participate in a study. One might expect that schools directors with an interest in healthy nutrition or overweight prevention might have been more willing to cooperate in the study. However, the percentage of schools that had a policy on overweight prevention was lower among the schools that completed the questionnaire than the schools that only returned the non-response card. Furthermore, non-response analyses showed that the prevalence of the presence of a soft drink vending machine or a vending machine containing sweets and candy bars at school did not differ between the schools that completed the questionnaire and schools that only returned the non-response card. Therefore, non response bias is not very likely.

Limitations are that the questionnaire was long and focused on overweight prevention only. Social desirable answering could have influenced our results. In particular among vocational education schools social desirable answering may have been a problem, because these schools seemed to be more aware of the overweight problem. However, because the current situation at vocational education school appeared to be less favourable than the situation at higher education schools we have no reasons to believe that the vocational education schools gave social desirable answers. For some questions the interpretation could differ between schools. For instance, the question on the school health policy. Although we additionally asked in the questionnaire whether the health policy was written down and included in the school rules, or whether the health policy was still in development, it is hard to judge the actual impact and implementation of the health policy.

The degree of urbanisation in the area around the school could not be accounted for. Degree of urbanisation could have an effect on some of the outcome variables, for instance the possibilities for the children to be physically active. It is not likely that the differences between school levels could be explained by differences in degree of urbanisation, because we have no indication that schools of a certain school level are more prevalent in more or less urban regions.

The questions on the curriculum were not included in this study, because all students in the Netherlands have to pass the same final exam for graduation, dependent on the school level. In addition, the curriculum differs between school levels, because the school levels prepare for different continuing educations and jobs.

### Comparison with other studies

Not many studies have described the school environment and the presence of a school policy on overweight prevention. A study in Belgium showed that 80% of the secondary school had vending machines[12] and in the USA similar findings have been reported[13]. The low number of schools with a policy on nutrition or on overweight prevention observed in this study is comparable with other countries[13,14]. The Belgium study also investigated the number of soft drink consumers by educational level and observed a higher number of consumers among students engaged in vocational education compared to students in general education[12].

### Interpretation of the results

Although our study does not have data on the actual intake of unhealthy foods and drinks by the students, literature shows that high availability of unhealthy foods and drinks at school are in general associated with an increased purchase and intake by the students[12,15-17]. Also, school policies aiming at the decrease in access of unhealthy foods have been shown to be associated with a less frequent purchase of these items among students[12]. The consumption of unhealthy foods like sugar-sweetened drinks and energy dense foods have been linked with an increased risk to develop overweight among children[18,19]. Given the high availability of unhealthy foods and drinks at schools and low number of schools that have a policy on nutrition or overweight prevention, schools are a promising setting for overweight prevention among Dutch adolescents[15]. These national findings are supportive for and in line with existing international approaches. The European Commission indicates the educational system to be a valuable environment for preventing overweight and the World Health Organization defines the school as a priority setting[20,21].

Schools should be encouraged to develop a school policy on healthy eating behaviour or, more specifically, on overweight prevention and to take actions to decrease the availability of unhealthy foods and drinks. Although only a small number of school reported to have a school policy on nutrition or on overweight prevention, our study shows that many schools have already taken some first steps to decrease the consumption of unhealthy foods and drinks and the stimulate healthy eating behaviour. This finding suggests that there is support in schools to play a role in the prevention of overweight.

Future studies with data on overweight status and foods intake among students are needed to study the actual influence of the school environment on overweight prevalence among students. Interestingly, the vocational education schools reported less often that overweight was less prevalent at their schools than among the children in the general population. This finding is in accordance with the higher overweight rates observed among students following vocational education reported in Dutch studies[10,11].

## Conclusions

In conclusion, this study describes the current situation at Dutch secondary schools and identifies principles for action. Currently, secondary schools are not actively involved in overweight prevention and the nutritional environment at most schools could be improved. A follow-up study is needed to follow the development of the obesogenity of the school environment and whether overweight prevention programmes in the secondary schools pay off. The small differences between school levels do not give reason for a differential approach for different school level or a specific focus at vocational education schools for overweight prevention. However, overweight is more prevalent among students at vocational education schools and vocational education schools are more aware of the overweight problem. Therefore these schools might be more motivated to implement overweight prevention strategies.

## Competing interests

The authors declare that they have no competing interests.

## Authors' contributions

All authors commented critically on the manuscript and agreed on the final version. SS analyzed the data and wrote the main part of the manuscript. LM designed the survey and collected the data. SR and GB helped design the survey, commented on the analysis and interpretation and reviewed drafts of the manuscript. WB originated and supervised the study.

## Pre-publication history

The pre-publication history for this paper can be accessed here:

http://www.biomedcentral.com/1471-2458/10/42/prepub
